# A comparison of biologicals in the treatment of adults with severe asthma – real-life experiences

**DOI:** 10.1186/s40733-020-00055-9

**Published:** 2020-05-13

**Authors:** Emma Kotisalmi, Auli Hakulinen, Mika Mäkelä, Sanna Toppila-Salmi, Paula Kauppi

**Affiliations:** 1 Respiratory Diseases and Allergology, University of Helsinki and Helsinki University Hospital, Inflammation Center, Meilahdentie 2, FI-00029 HUS, P.O. Box 160, Helsinki, Finland; 2 Respiratory Diseases and Allergology, University of Helsinki and Helsinki University Hospital, Heart and Lung Center, Helsinki, Finland; 3 Otorhinolaryngology, University of Helsinki and Helsinki University Hospital, Inflammation Center, Helsinki, Finland

**Keywords:** Anti-IgE, Anti-IL5, Asthma, Biological therapy, Corticosteroid, Eosinophils, Exacerbation, IgE, Chronic rhinosinusitis

## Abstract

**Background:**

Anti-IgE (omalizumab) and anti-IL5/IL5R (reslizumab, mepolizumab and benralizumab) treatments are available for severe allergic and eosinophilic asthma. In these patients, studies have shown beneficial effects in oral corticosteroid use and exacerbations. The aim of this retrospective single-center study was to evaluate the effect of biological therapy on severe asthma and to compare different therapies.

**Methods:**

We collected and analysed results of anti-IL5/IL5R and anti-IgE therapies for asthma from January 2009 until October 2019 in specialized care. We compared number of exacerbations, asthma symptoms and use of per oral corticosteroids and antimicrobics because of asthma before and during biological therapy, and in a separate analysis need for per oral corticosteroids, antimicrobics or surgery due to upper respiratory tract diseases in asthmatics receiving biologicals. The analyses were done using the Chi square test, T-test or Mann-Whitney U -test, the Kruskall-Wallis test or the Wilcoxon test.

**Results:**

Of 64 patients, 40 used continuous per oral corticosteroid therapy prior to biological therapy. The mean daily dose of per oral corticosteroid was reduced in those with anti-IL5/IL5R therapy (− 3.0 mg, *p* = 0.02). The number of annual per oral corticosteroid courses decreased in both the anti-IL5/IL5R (− 2.8 courses, *p* < 0.05) and anti-IgE groups (− 1.3 courses, *p* < 0.05). The number of annual antibiotic courses (− 0.7 courses, *p* = 0.04) and total number of exacerbation events (− 4.4 events/year, *p* < 0.05) were reduced in the anti-IL5/IL5R group. In the 55 asthma patients analysed for upper respiratory tract findings, the results suggested a reduction in need for chronic rhinosinusitis surgery during biological therapy.

**Conclusions:**

Results with biological therapies in this real-life clinical setting are comparable to those reported in clinical trials. Biological therapy reduces exacerbations and per oral corticosteroid use.

**Trial registration:**

NCT04158050, retrospectively registered 6.11.2019.

## Background

Asthma is a common non-communicable disease with over 300 million people affected worldwide. The proportion of severe asthma of all asthmatics is 5–10% [1]. The GINA (Global Initiative for Asthma) guideline defines severe asthma as a condition that requires GINA step 4 or 5 treatment to be controlled or becomes uncontrolled due to a reduction in this ongoing high dose treatment [2]. Uncontrolled asthma is characterised by poor symptom control (frequent symptoms or need of short acting beta agonists, symptoms at night, restricted activity due to asthma) and/or frequent exacerbations (two or more exacerbations requiring per oral corticosteroid (OCS) within a year or 1 or more exacerbations leading to hospitalisation within a year) [2–4].

Important target molecules of biological therapies of severe asthma in use today are the immunoglobulin E (IgE) molecule and the interleukin-5 (IL5) and IL5 receptor (IL5R) molecules [5]. Omalizumab is an anti-IgE antibody and treatment criteria include severe allergic asthma and elevated serum IgE level and at least one positive skin prick test to an aeroallergen, or elevated specific aeroallergen IgE levels [6]. In the uncontrolled severe allergic asthma patients, omalizumab combined with high dose combination therapy has reduced exacerbations by 25–35%, reduced the use of OCS and symptoms and improved lung function and quality of life [6–11]. Mepolizumab, reslizumab and benralizumab are anti-IL5 and anti-IL5R-drugs that reduce exacerbations and OCS use in severe eosinophilic asthma and improve the quality of life with little effect on lung function [8–10, 12–17].

The unified airway theory suggests that upper and lower airways function as a unit, and that similar inflammatory processes occur in different parts of the respiratory tract [18, 19]. In chronic rhinosinusitis with nasal polyposis (CRSwNP), the inflammatory reaction, like in severe allergic and eosinophilic asthma, is of Th2 type and includes eosinophils [20, 21].

Previous literature suggest that 50% of patients suffering from severe asthma also suffer from chronic rhinosinusitis (CRS) or nasal polyposis [22]. Increased asthma severity has also been linked to a greater prevalence of nasal polyposis [18]. Anti-IL5 (reslizumab and mepolizumab) therapies used in severe eosinophilic asthma have improved the nasal polyp score in patients suffering from severe nasal polyposis being refractory to corticosteroid therapy. Anti-IgE therapy has improved the nasal polyp score in patients with severe comorbid asthma [20, 23].

The aim of this retrospective real-life study was to determine if Finnish patients receiving biological therapy for severe asthma benefit from the treatment due to reduction in exacerbations, OCS dose and courses of OCS and antibiotics in a real-life setting with a broader population than in a randomised controlled trial, and to compare anti-IL5/IL5R therapy results with anti-IgE therapy results in a real-life setting. We also aim to describe the impact of biological therapy on upper respiratory tract findings and need for treatment in asthma patients receiving biologicals.

## Material and methods

### Patients and study design

This is a retrospective clinical study on adult patients (18 years or more) undergoing biological therapy for severe asthma at a university central hospital in Finland. This is a real-life study with a broader patient population than in a randomised controlled trial. The data was collected from electronic medical records from between January 2009 and October 2019. Omalizumab has been used for the treatment of asthma in this university hospital since January 2009, mepolizumab since April 2016, reslizumab since February 2017 and benralizumab since October 2018. In Finland and the university hospital involved in this study, biological therapy is considered for patients with asthma that remains uncontrolled despite high doses of inhaled corticosteroid (ICS) and at least one other controller medication and need for continuous OCS or contraindications (or clinically significant side effects of OCS) against OCS and/or frequent courses of OCS and reduced lung function [12, 13, 24].

Omalizumab was chosen if the patient had perennial allergy and their weight and S-IgE were in accordance with the indications for taking omalizumab [6]. Reslizumab, mepolizumab or benralizumab were chosen in eosinophilic asthma and the patients were categorised to receive either of the anti-IL5-therapies in turn [12, 13, 24]. All the patients included in this study were systematically assessed at the severe asthma clinic in the university hospital after the management of comorbidities and potential adherence issues and were defined as suffering from severe asthma and needed a biological add-on therapy to control exacerbations, based on the hospital’s criteria for biological therapy described above. The decision for initiating treatment was made by an evaluation group of four pulmonology specialists, one paediatric allergologist and two rhino-allergologists.

All asthma patients receiving biological therapy were evaluated at three to 4 months after the initiation of therapy, and regularly thereafter at the university hospital. By protocol, the patients should also have visited an Otorhinolaryngologist before and during the biological therapy**.** The treatment response was evaluated at every physician appointment. The symptoms, laboratory tests and spirometry results, exacerbations, maintenance medication and additional corticosteroids or treatments with antibiotics were documented. If there was no treatment response, the biological therapy was discontinued.

For each patient included in the study, we collected the following data: gender, age, smoking status, pulmonary function test results (spirometry), blood eosinophil count, skin prick test or aeroallergen specific serum IgE positivity, serum total IgE, exhaled nitric oxide level, associated upper respiratory tract diseases (chronic and allergic rhinosinusitis, nasal polyposis), other comorbidities, osteoporosis, pulmonary imaging results, asthma control test (ACT) score, exacerbations and associated medical treatment, sick-leave due to asthma and hospitalization and emergency room visits due to asthma before and during biological therapy. The data during the biological therapy represent the latest parameters in October 2019. A course of per oral corticosteroid treatment was defined as at least a doubling of the previous per oral corticosteroid dose for at least 3 days. Antibiotic treatment was documented, if it was due to respiratory infection and asthma exacerbation.

We also documented upper respiratory tract findings and the need for CRS- surgery or other treatment (antibiotics and corticosteroids) of upper respiratory tract diseases of asthma patients before and during the biological therapy. We collected data concerning corticosteroid and antibiotic treatment due to upper respiratory tract findings (rhinosinusitis, otitis, nasal polyposis and bronchitis) and the need for CRS-surgery. We collected data representing the 12 months before and during biological therapy, and the data during therapy represent the latest parameters in October 2019.

In this study, forced expiratory volume in 1 sec (FEV1) and forced vital capacity (FVC) were reported in litres instead of percentages of predicted values because of a change in spirometry reference values in Finland during this study [25]. According to this change, the previous spirometry values are reported as litres and percentages of predicted values and the newest spirometry values are reported as litres and Z-scores [25]. Since the reference values have been updated, the percentages of predicted values in the beginning of the evaluation are not comparable to the percentages of predicted values in 2019 (at the time of analysis) [25]. Thus, here we report the spirometry results in litres.

A research approval was obtained from the research board of the university hospital before the research was started. In addition, the study was registered in the clinical trials. The research questions and outcome measures were developed according to scientific literature. The normal treatment protocol for biological therapies in asthma in the hospital district was used in this study. The research did not form any additional burden to the patients involved. Since this was a retrospective study, no patient consent form was required.

### Statistical analysis

In this real-life study, we compared the data above before and during treatment of severe asthma with biological therapy. Patients receiving reslizumab, mepolizumab or benralizumab were grouped into an anti-IL5/IL5R group, and patients receiving omalizumab formed an anti-IgE group. Patients receiving anti-IL5 or anti-IL5R therapy were analysed both separately and all together. The same analyses were done for patients receiving anti-IgE therapy. The endpoint data were collected between August and October 2019 and the time interval for comparison was the last 12 months before biological therapy and the latest 12 months during biological therapy. If the time of use of biologicals was shorter than 12 months, the data was adjusted to meet a 12-month follow-up both in the asthma analysis and in the analysis of upper respiratory tract findings. In the analysis, the per oral corticosteroid dose and the annual number of courses of per oral corticosteroid treatment, courses of antibiotics, hospitalisations, emergency room visits, sick leaves due to asthma and total number of exacerbation events (defined as the sum of the previous events) before and during biological therapy were compared using the Chi square test, T-test or Mann-Whitney U -test, the Kruskall-Wallis test or the Wilcoxon test. The statistical test was chosen based on whether the variable was continuous or discrete and whether a continuous variable was normally distributed or not. A *p*-value 0.05 was set as the level of significance.

Finally, we also analysed the impacts of anti-IL5/IL5R and anti-IgE therapy on upper airway findings (nasal polyposis and mucous swelling at inspection (yes or no)) and the need for treatment (per oral corticosteroids, antibiotics or CRS-surgery) in asthma patients receiving biologicals. The analyses were done using the statistical tests described above.

## Results

Altogether 64 asthma patients were chosen to be treated with biological therapy in this university central hospital between January 2009 and October 2019. The 64 patients receiving biological therapy were included in this study. Twenty-two patients received omalizumab (mean age 48 years, 68% women) and 42 received anti-IL5 or anti-IL5R therapy (mean age 56 years, 55% women). Of these 42, 13 received reslizumab (mean age 56 years, 46% women), 24 received mepolizumab (mean age 58 years, 58% women) and 5 received benralizumab (mean age 48 years, 60% women) (Table 1 and Additional file 1 table 1). None of the patients smoked regularly.

**Table 1:** Clinical characteristics and co-morbidity of asthma patients before anti-IL5/IL5R and anti-IgE therapy

Characteristics	Anti-IL5/IL5R, ***n*** = 42	Anti-IgE, ***n*** = 22
	**Reslizumab, Mepolizumab, Benralizumab**	**Omalizumab**
**Time of use (months)**	12.79 (3–29. SD 8.49)	49.41 (6–127. SD 35.34)
**Age (years)**	56 (32–73. SD 9.75)	48 (28–76. SD 10.67)
**Women (number, (%))**	23 (55)	15 (68)
**Smokers (number, (%))**	0 (0)	0 (0)
**Ex-smokers (number, (%))**	12 (29)	2 (9)
**Body mass index (kg/m**^**2**^**)**	28.9 (16.9–39. SD 5.1)	27.7 (20.4–36.8. SD 4.1)
**Nasal polyposis (number, (%))**	31 (74)	12 (55)
**Chronic rhinosinusitis (CRSwNP or CRSsNP) (number, (%))**	39 (93)	18 (82)
**Allergic rhinitis (number, (%))**	12 (29)	17 (77)
**ASA intolerance (number, (%))**	9 (21)	5 (23)
**Osteoporosis (number, (%))**	16 (38)	8 (36)
**Hypertension (number, (%))**	20 (48)	8 (36)
**Diabetes mellitus (number, (%))**	3 (7)	3 (14)
**Hypothyroidism (number, (%))**	7 (17)	3 (14)
**Coronary artery disease (number, (%))**	1 (2)	0 (0)
**Gastroesophageal reflux disease (number, (%))**	10 (24)	10 (45)
**Atrial fibrillation (number, (%))**	3 (7)	1 (5)
**Positive skin prick test or elevated allergen specific serum IgE (number, (%))**	17 (40)	22 (100)
**Pathological HRCT findings (number, (%))**^**a**^	30 (71)	14 (64)
**Patients with daily use of OCS (number, (%))**^**b**^	30 (71)	10 (45)
**Mean daily OCS dose before treatment (mg)**	7.07 (0–40. SD 7.13)	4.82 (0–20. SD 6.50)
**Courses of OCS before treatment**^**c**^	4.21 (0–12. SD 2.75)	2.50 (0–6. SD 1.56)
**Courses of antibiotics before treatment**^**c**^	1.40 (0–5. SD 1.42)	0.95 (0–3. SD 0.93)
**Emergency room visits before treatment**^**c**^	0.62 (0–8. SD 1.51)	0.18 (0–1. SD 0.39)
**Sick leaves before treatment**^**c**^	0.93 (0–6. SD 1.72)	0.59 (0–4. SD 1.03)
**Hospitalisations before treatment**^**c**^	0.52 (0–8. SD 1.42)	0.45 (0–2. SD 0.66)
**FEV1 (mean) before treatment (litres)**	2.27 (0.99–4.18. SD 0.78)	2.83 (1.5–4.2. SD 0.86)
**FVC (mean) before treatment (litres)**	3.38 (1.71–5.25. SD 0.99)	3.82 (2.1–5.8. SD 0.85)
**FEV1/FVC (mean) before treatment**	0.67 (0.42–0.91. SD 0.12)	0.74 (0.48–0.91. SD 0.13)
**Blood eosinophil count (mean) before treatment (E9/litre)**	0.45 (0.03–1.84. SD 0.37)	0.43 (0–1.34. SD 0.41)
**Exhaled nitric oxide (mean) (ppb)**	28.17 (5–110. SD 28.98)	35.66 (5–123. SD 36.30)
**Serum total IgE (mean) (kU/litre)**	290.85 (8–4672. SD 786.18)	313.36 (14–720. SD 248.76)
**ACT (mean)**^**d**^	15.6 (8–23. SD 4.28)	13.4 (7–22. SD 4.82)

^a^defined as HRCT (high resolution computed tomography) detected ground glass pattern, bronchiectasis, mucous plugs, atelectasis, nodularity or bronchial thickening

^b^OCS = per oral corticosteroids

^c^Number due to asthma during the past 12 months before biological treatment initiation

^d^ACT = Asthma control test, maximal score 25 points

Mean time of use (at analysis) of biological therapy was 12.8 months in the anti-IL5/IL5R group and 49.4 months in the anti-IgE group. The time of use was calculated from the initiation date of the biological therapy to the date of analysis in October 2019. Anti-IL5/IL5R therapy was discontinued in 9 patients (2 patients receiving reslizumab, 6 patients receiving mepolizumab and 1 patient receiving benralizumab) and anti-IgE therapy was discontinued in 5 patients (Table 2 in Additional file 1).

The therapy was discontinued due to infections (*n* = 2), rise in transaminases (*n* = 1), dyspnoea after injection (*n* = 1), recurrent gouts (*n* = 1) or lack of response. The mean point of discontinuation of therapy in patients with no response was 6.7 months in the anti-IL5/IL5R group and 14.3 months in the anti-IgE group. One patient became pregnant during omalizumab therapy. Other reported side effects were headache, transient fatigue after injection, musculoskeletal aches, transient fever after injection, nausea and worsening of lower limb pitting oedema. (Table 2 in Additional file 1) Patients with discontinued biological therapy were included in the analyses. All the other patients continued receiving biologicals after the date of analysis.

All the asthma patients in the anti-IL5/IL5R group, except one, used high dose ICS as controller medication before receiving biological therapy. One patient receiving mepolizumab used an intermediate dose of ICS. In the anti-IgE group, 20 patients (91%) used a high dose of ICS and 2 patients (9%) an intermediate dose of ICS before initiation of biologicals. Before biological therapy, 41 patients (98%) in the anti-IL5/IL5R group used long acting beta agonist (LABA) daily and 35 patients (83%) used two or more asthma controllers, in addition to ICS, on a daily basis. The corresponding proportions for the anti-IgE group was 19 patients (86%) for daily LABA and 18 patients (82%) for two or more additional asthma controllers. In 30 patients (71%) in the anti-IL5/IL5R group and in 10 patients (45%) in the anti-IgE group asthma treatment involved continuous OCS prior to biological therapy. (Table 2 and Additional file 1 table 3).

**Table 2:** Controller medication of asthma patients before anti-IL5/IL5R and anti-IgE therapy

Controller medication before biologicals	Anti-IL5/IL5R (reslizumab, mepolizumab or benralizumab), ***n*** = 42	Anti-IgE (omalizumab), ***n*** = 22
**ICS daily dose (number, (%))**^**a**^		
**High**	41 (98)	20 (91)
**Intermediate**	1 (2)	2 (9)
**Low**	0 (0)	0 (0)
**Daily use of long acting beta-agonist (number, (%))**	41 (98)	19 (86)
**Daily use of theophylline (number, (%))**^**b**^	7 (17)	7 (32)
**Daily use of montelukast (number, (%))**^**c**^	30 (71)	13 (59)
**Daily use of long-acting anticholinergic (number, (%))**	27 (64)	15 (68)
**Daily use of one asthma controller medication in addition to ICS (number, (%))**	7 (17)	4 (18)
**Daily use of two or more asthma controller medications in addition to ICS (number, (%))**	35 (83)	18 (82)
**Daily use of OCS (number, (%))**	30 (71)	10 (45)

^a^ICS = inhaled corticosteroid. For beclomethasone and budesonide: low dose 0–399 μg/day, intermediate dose 400–799 μg/day and high dose 800 μg/day or more, for fluticasone low dose 0–249 μg/day, intermediate dose 250–499 μg/day and high dose 500 μg/day or more, and for ciclesonide low dose 0–159 μg/day, intermediate dose 160–319 μg/day and high dose 320 μg/day or more. The ICS daily dose was defined as the dose prior to initiation of biological therapy

^b^Additionally, 3 patients in the reslizumab group, 5 patients in the mepolizumab group and 3 patients in the omalizumab group had received theophylline earlier but discontinued it because of lack of response or side effects

^c^Additionally, 1 patient in the reslizumab group, 3 patients in the mepolizumab group and 6 patients in the omalizumab group had received montelukast earlier but discontinued it because of lack of response or side effects

Before initiating biologicals, the mean daily OCS dose was 7.1 mg in the anti-IL5/ILR5 group and 4.8 mg in the anti-IgE group, and the frequency of OCS courses was 4.2/year in the anti-IL5/IL5R group and 2.5/year in the anti-IgE group. (Table 1 and Additional file 1 table 1).

Of patients receiving anti-IL5/IL5R therapy 39 (93%) suffered from CRS. Of them, 79% had CRS with nasal polyposis (CRSwNP) and 21% had CRS without nasal polyposis (CRSsNP). 77% of the patients in the anti-IgE group suffered from allergic rhinitis. 38% of patients treated with any biological suffered from osteoporosis (Table 1). Among patients receiving any of the four biologicals, 69% had abnormal findings in high resolution computed tomography (HRCT) (Table 4 in Additional file 1). The mean serum total IgE and exhaled nitric oxide levels at baseline were higher in the anti-IgE group than in the anti-Il5/IL5R group, but the difference was not statistically significant (Table 1).

**Fig. 1:**
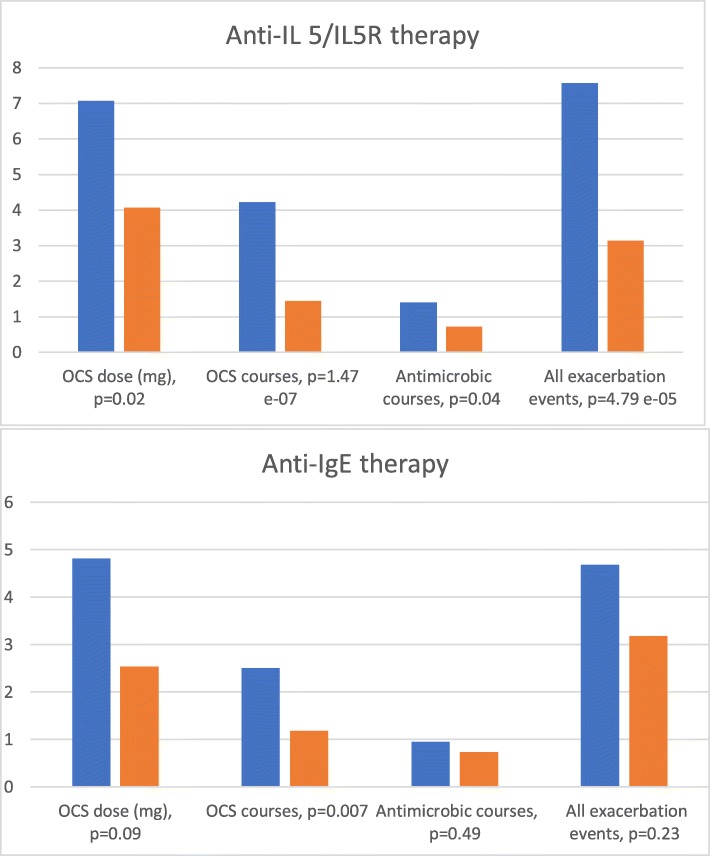
Treatment results of Anti-IL5/IL5R therapy (reslizumab, mepolizumab or benralizumab) and anti-IgE therapy (omalizumab) in patients suffering from severe asthma. Oral corticosteroid (OCS) courses, antibiotic courses and all exacerbation events are presented as number during the last 12 months preceding biological therapy (blue columns) and the latest 12 months during biological therapy (orange columns). Patients with discontinued biological therapy were included in the analyses

During biological therapy, the mean daily OCS dose was significantly reduced in the anti-IL5/IL5R group: the mean daily dose during therapy was 4.1 mg (*p* = 0.020). A reduction in the OCS daily dose was also seen in the omalizumab group (daily OCS dose during therapy was 2.5 mg), but the reduction was not statistically significant (*p* = 0.085). The frequency of OCS courses during biological therapy reduced significantly in both groups, with − 2.8 courses/year in the anti-IL5/IL5R group (*p* < 0.05) and − 1.3 courses/year in the anti-IgE group (*p* < 0.05) (Table 3, Fig. 1 and Additional file 1 table 5).

**Table 3:** Treatment response in asthma patients with anti-IL 5/IL5R (reslizumab, mepolizumab or benralizumab) therapy and anti-IgE (omalizumab) therapy. Patients with discontinued biological therapy were included in the analyses

	Anti-IL5/IL5R (reslizumab, mepolizumab, benralizumab)	***N*** = 42		Anti-IgE (omalizumab)	***N*** = 22	
	Baseline	Change	P	Baseline	Change	P
**Mean daily OCS dose (mg)**^**a**^	7.07 (0–40. SD 7.13)	−3.00 (−35–25. SD 7.91)	0.020	4.82 (0–20. SD 6.50)	−2.29 (−20–0. SD 5.80)	0.085
**Courses of OCS**^**b**^	4.21 (0–12. SD 2.75)	−2.77 (−7.64–1. SD 2.81)	1.47 e^−07^	2.50 (0–6. SD 1.56)	−1.32 (−4–10. SD 2.01)	0.007
**Courses of antibiotics**^**b**^	1.40 (0–5. SD 1.42)	−0.69 (−5–7. SD 2.08)	0.040	0.95 (0–3. SD 0.93)	− 0.23 (− 3–5. SD 1.47)	0.488
**Emergency room visits**^**b**^	0.62 (0–8. SD 1.63)	−0.29 (−6–4. SD 1.30)	0.159	0.18 (0–1. SD 0.39)	0.18 (− 1–5. SD 1.19)	0.492
**Hospitalisations**^**b**^	0.52 (0–8. SD 1.42)	− 0.20 (−8–4. SD 1.63)	0.448	0.45 (0–2. SD 0.66)	0.00 (− 1–5. SD 1.21)	1.00
**Sick leaves**^**b**^	0.93 (0–6. SD 1.72)	− 0.58 (− 6–6. SD 1.86)	0.053	0.59 (0–4. SD 1.03)	−0.14 (− 3–11. SD 1.18)	0.602
**Total number of exacerbation events**^**c**^	7.57 (1–26. SD 5.59)	−4.43 (− 22–13. SD 6.24)	4.79 e^− 05^	4.68 (0–11. SD 3.40)	−1.50 (− 7–21. SD 5.55)	0.229
**Blood eosinophil count (E9/litre)**	0.45 (0.03–1.84. SD 0.37)	− 0.39 (− 1.79–0.01. SD 0.38)	2.36 e^− 07^	0.43 (0–1.34. SD 0.41)	−0.08 (− 0.61–0.52. SD 0.41)	0.433
**Mean FEV1 (litres)**	2.27 (1.0–4.2. SD 0.78)	0.17 (− 0.5–1.3. SD 0.41)	0.014	2.83 (1.5–4.2. SD 0.86)	0.05 (− 0.6–1.3. SD 0.54)	0.720
**ACT (mean)**^**d**^	15.63 (8–23. SD 4.28)	4.46 (− 15–13. SD 5.81)	0.0005	13.40 (7–22. SD 4.82)	8.50 (2–17. SD 5.63)	0.015
**Exhaled nitric oxide (mean) (ppb)**	28.17 (5–110. SD 28.98)	− 4.78 (− 68–40.1. SD 18.83)	0.759	35.66 (5–123. SD 36.30)	− 12.63 (− 71–21.5. SD 36.56)	0.592
**Serum total IgE (mean) (kU/litre)**^**e**^	290.85 (8–4672. SD 786.18)	160 (− 304–1693. SD 551.61)	0.424	313.36 (14–845. SD 248.76)	77.83 (− 24–369. SD 133.38)	0.249

^a^OCS = per oral corticosteroid

^b^Number due to asthma during the last 12 months

^c^defined as the sum of courses of oral corticosteroid and antimicrobial drugs, sick leaves, hospitalisations and emergency room visits due to asthma during the last 12 months

^d^ACT = Asthma control test, maximal score 25 points

^e^In the hospital district that this study was conducted, serum total IgE is systematically measured before initiation of biological therapy but not during therapy. As a result, serum total IgE levels during therapy were available only occasionally and the change in the serum IgE level cannot be used as treatment effect measurement

The annual number of courses of antibiotics was reduced from 1.4 to 0.7 (*p* = 0.04) in the anti-IL5/IL5R group and from 1.0 to 0.7 (*p* = 0.488) in the anti-IgE group (Table 3 and Additional file 1 table 5, Fig. 1). The total annual exacerbation events (defined as the sum of courses of oral corticosteroid and antibiotics, sick leaves, hospitalisations and emergency room visits because of asthma during the last 12 months) were significantly reduced in the anti-IL5/IL5R group from 7.6/year to 3.2/year (p < 0.05). The reduction in total exacerbation events was not statistically significant in the anti-IgE group (from 4.7/year to 3.2/year, *p* = 0.229) (Table 3 and Additional file 1 table 5, Fig. 1). In both therapy groups, the asthma control test (ACT) scores improved significantly (+ 4.5 scores in the anti-IL5/IL5R group and + 8.5 scores in the anti-IgE group).

Altogether 55 asthma patients were included in the upper respiratory tract analysis, 38 patients receiving anti-IL5/IL5R therapy (mean age 54 years, 47% women) and 17 patients receiving anti-IgE therapy (mean age 48 years, 71% women). In this analysis, we included three patients in whom the indication for anti-IL5/IL5R therapy was nasal polyposis instead of asthma. These 3 patients also suffered from asthma**.** Twelve patients were excluded from the analysis due to insufficient data. In the anti-IL5/IL5R group and the anti-IgE group, 89 and 88% suffered from CRS, respectively, of which 85 and 73% suffered from CRSwNP. At baseline, 89% of patients in the anti-IL5/IL5R group and 82% of patients in the anti-IgE group used daily intranasal corticosteroids (Table 4 and Additional file 1 table 6).

**Table 4:** Upper respiratory tract and other characteristics before initiation of anti-IL5/IL5R or anti-IgE treatment. The time of use describes mean time of use of biological therapy in months

Characteristics	Anti-IL5/IL5R	Anti-IgE
	Reslizumab, Mepolizumab, Benralizumab, ***n*** = 38	Omalizumab, ***n*** = 17
**Asthma (number, (%))**	38 (100)	17 (100)
**Nasal polyposis as only indication for biological therapy (number, (%))**	3 (8)	0 (0)
**Time of use (months)**^**a**^	12.4 (1–29. SD 7.6)	43.8 (5–109. SD 35.6)
**Age at analysis (years)**	54.2 (25–72. SD 10.8)	47.6 (28–76. SD 11.5)
**Women (number, (%))**	18 (47)	12 (71)
**BMI**^**b**^**(kg/m2)**	28.1 (16.9–38.8. SD 4.8)	27.6 (23–35. SD 3.3)
**Use of intranasal corticosteroids (number, (%))**	34 (89)	14 (82)
**Use of oral antihistamine (number, (%))**	13 (34)	8 (47)
**Use of montelukast (number, (%))**	27 (71)	9 (53)
**Positive skin prick test or allergen specific serum IgE (number, (%))**	16 (42)	17 (100)
**Smokers (number, (%))**	1 (3)	0 (0)
**Ex-smokers**^**c**^**(number, (%))**	9 (24)	2 (12)
**Nasal polyposis (number, (%))**	29 (76)	11 (65)
**Chronic rhinosinusitis (CRSwNP or CRSsNP) (number, (%))**	34 (89)	15 (88)
**ASA intolerance (number, (%))**	9 (24)	3 (18)
**Osteoporosis (number, (%))**	12 (32)	4 (24)
**Blood eosinophil count (E9/l)**	0.48 (0.00–1.84. SD 0.38)	0.50 (0.01–1.21. SD 0.42)

^a^Time of use of biological therapy

^b^*BMI* = Body mass index

^c^Defined as previous daily smoking at minimum 1 year

The annual frequency of OCS courses due to upper respiratory tract diseases in asthmatics receiving biologicals reduced in both therapy groups, but no reduction was seen in antibiotic courses. With biological therapy the need for CRS-surgery reduced statistically significantly, but the absolute numbers were low. The occurrence of nasal polyps and mucosal oedema in intranasal inspection was significantly reduced in the anti-IL5/IL5R group (*p* = 0.038 and *p* = 0.004 respectively) but not in the anti-IgE group (Table 5).

**Table 5:** Impacts of anti-IL5/IL5R and anti-IgE therapy on upper respiratory tract findings and need for treatment in asthma patients receiving biologicals. The parameters were adjusted to match a 12-month period of biological therapy. Patients with discontinued biological therapy were included in the analyses

	Anti-IL5/IL5R (Reslizumab, Mepolizumab, Benralizumab)	***N*** = 38		Anti-IgE (Omalizumab)	***N*** = 17	
	Baseline	Change	P	Baseline	Change	P
**Courses of OCS (number/year)**^**a**^	1.24 (0–8. SD 2.03)	−0.79 (−8–2. SD 2.14)	0.031	0.82 (0–3. SD 1.04)	−0.59 (−3–1. SD 1.03)	0.037
**Courses of antibiotics (number/year)**	0.94 (0–5. SD 1.26)	0.12 (−4–5. SD 1.99)	0.718	1.12 (0–4. SD 1.23)	−0.22 (− 3–7. SD 2.09)	0.674
**Polypectomy (number)**	1.37 (0–20. SD 3.29)	−1.24 (−20–1. SD 3.30)	0.028	1.59 (0–20. SD 4.67)	−1.41 (− 20–1. SD 4.69)	0.246
**Ethmoidectomy (number)**	0.76 (0–4. SD 0.87)	−0.71 (− 4–1. SD 0.94)	5.10 e^− 05^	0.29 (0–1. SD 0.46)	−0.12 (− 1–2. SD 0.68)	0.496
**FESS (number)**^**b**^	1.03 (0–4. SD 0.87)	− 0.95 (− 3–1. SD 0.89)	1.34 e^− 07^	1.12 (0–4. SD 1.13)	−0.82 (− 4–1. SD 1.25)	0.018
**Polyps in nasal inspection (number, (%))**	22 (58)	−9	0.038	7 (41)	−1	0.724
**Mucous oedema in nasal inspection (number, (%))**	19 (50)	−12	0.004	7 (41)	+ 4	0.169

^a^OCS = per oral corticosteroid

^b^FESS = Functional endoscopic sinus surgery

## Discussion

This study is the first of its kind among research on biological therapy, which compares different treatment groups in a real-life setting. This study suggests that anti-IL5/IL5R therapy reduces exacerbations and anti-IL5/IL5R and anti-IgE therapies reduce per oral corticosteroid use.

As other studies, also this study shows that patients suffering from severe asthma have several exacerbations annually [26]. In previous research reports, both IL5 antibodies and omalizumab have reduced exacerbation rates, need of periodic OCS and daily dose of OCS [7, 8, 13, 18, 27, 28]. Results of this study are otherwise in concordance with previous studies, except from the lack of statistical significance in the reduction of the daily OCS dose and total annual exacerbation events with omalizumab.

In a previous indirect comparison, the treatment results with omalizumab and mepolizumab were considered similar and no superiority of a single anti-IL5 therapy has been reported from indirect comparisons between reslizumab, mepolizumab and benralizumab [29]**.** In the comparison, mepolizumab reduced exacerbation rates a little more than omalizumab, but the finding was not statistically significant [30]. In our comparison, we also calculated treatment results separately for reslizumab, mepolizumab and benralizumab (Table 5 in Additional file 1). Reslizumab and mepolizumab both reduced total exacerbations significantly, but the daily OCS dose and frequency of antibiotic courses was reduced significantly only in the reslizumab group. The benralizumab group was very small (*n* = 5), and the results with benralizumab can’t therefore be reliably interpreted.

Literature suggests improved life quality with biological therapy in severe asthma [6, 8–11, 13–15, 18, 31]. In our study, we detected a significant improvement in ACT scores with both anti-IL5 and anti-IgE therapy. Interestingly, according to ACT scores, the anti-IgE and anti-IL5/IL5R groups seemed to be equally symptomatic at baseline, despite that a lower daily OCS dose and fewer courses of OCS and other exacerbation events at baseline suggested a less severe disease in the anti-IgE group compared to the anti-IL5/IL5R group. It can further be discussed, whether lower OCS dose, fewer OCS and antibiotic courses and fewer total exacerbation events at baseline explain milder treatment results with anti-IgE therapy compared to anti-IL5/IL5R therapy.

Studies have also suggested, that type 2 inflammation response and asthma can be expressed as a combination of IgE mediated allergic asthma and eosinophilia [9, 32, 33]. In our study the allergic and eosinophilic phenotypes were also overlapping. Forty percent of patients receiving IL5 or IL5R antibodies had at least one positive result on skin prick tests or elevated allergen specific serum IgE levels. Further, the mean blood eosinophil count (B-eos) among patients receiving omalizumab was 0.4 x 10e9/l and earlier the suggested cut-off value for B-eos in the eosinophilic phenotype has varied from 0.15 to 0.4 x 10e9/l [34].

Nasal polyps and use of montelukast were more common in the anti-IL5/IL5R group than in the anti-IgE group. Nasal polyps might be a marker of more significant treatment effect of anti-IL5 therapy when compared to eosinophilic asthma without nasal polyps. On the other hand, rather small study groups may impact on the results. Among 55 asthma patients analysed for upper respiratory tract findings and need of treatment, the need of CRS-surgery seemed to reduce in patients receiving biological therapy, a finding in concordance with an RCT conducted in 2017 [35]. The impact of biologicals on need of surgical procedures and presence of polyps and mucosal oedema in patients with comorbid severe asthma seemed to be milder in the anti-IgE group compared to patients receiving anti-IL5/IL5R therapy. We must though remember that in this study, the number of surgical procedures was low. Also, difficulties to differ upper respiratory tract symptoms from asthma symptoms may bias the calculation of OCS courses prescribed for upper respiratory tract diseases in asthmatics receiving biologicals. More real-life studies are needed.

The rather small size of the study population is a limitation of this study and should be taken into consideration while interpreting the results, as should the retrospective design of this study.

It seems likely that in future treatment of severe asthma will be even more individualised and that there will be more biological treatment options available. Other biological therapies studied for treatment of severe asthma with promising results are e.g. pitrakinra (anti-IL 4), dupilumab (anti-IL 4 alpha) and tezepelumab (anti-TSLP; thymic stromal lymphopoietin) [36, 37]. Of these, dupilumab has recently received indication for treatment of nasal polyps [38].

## Conclusions

Patients suffering from severe asthma are symptomatic and have exacerbations despite of OCS therapy. Most of the patients suffer from chronic or allergic rhinitis or nasal polyposis. More than one third of patients suffer from osteoporosis at a relatively early age. In this retrospective real-life study, biologicals reduced OCS courses in both groups and reduced total number of exacerbation events in the anti-IL5/IL5R group, and CRS-surgery rate in patients with co-morbid CRS. We still need more data, on for how long biological therapy should be continued and on whether the treatment results are altered by a possible temporary discontinuation of the therapy. More real-life studies with increased population size are needed.

## Supplementary information

**Additional file 1.** Contains supporting tables on the study results. The tables are numbered table 1 to 5.

## Data Availability

The datasets generated and/or analysed during the current study are not publicly available due to privacy or ethical restrictions but are available from the corresponding author on reasonable request.

## Abbreviations

ACT: Asthma control test
BMI: Body mass index
CRS: Chronic rhinosinusitis
CRSwNP: Chronic rhinosinusitis with nasal polyposis
CRSsNP: Chronic rhinosinusitis without nasal polyposis
FESS: Functional endoscopic sinus surgery
FEV1: Forced expiratory volume in 1 sec
FVC: Forced vital capacity
GINA: Global Initiative for Asthma
HRCT: High resolution computed tomography
ICS: Inhaled corticosteroid
IgE: Immunoglobulin E
IL5: Interleukin 5
IL5R: Interleukin 5 receptor
LABA: Long acting beta agonist
OCS: Oral corticosteroid
RCT: Randomized controlled trial
TSLP: Thymic stromal lymphopoietin
