# Correction to: A comparison of biologicals in the treatment of adults with severe asthma – real-life experiences

**DOI:** 10.1186/s40733-020-00063-9

**Published:** 2020-10-02

**Authors:** Emma Kotisalmi, Auli Hakulinen, Mika Mäkelä, Sanna Toppila-Salmi, Paula Kauppi

**Affiliations:** 1 Respiratory Diseases and Allergology, University of Helsinki and Helsinki University Hospital, Inflammation Center, Meilahdentie 2, FI-00029 HUS, P.O. Box 160, Helsinki, Finland; 2 Respiratory Diseases and Allergology, University of Helsinki and Helsinki University Hospital, Heart and Lung Center, Helsinki, Finland; 3 Otorhinolaryngology, University of Helsinki and Helsinki University Hospital, Inflammation Center, Helsinki, Finland

**Correction to: Asthma Res Pract 6, 2 (2020)**

**https://doi.org/10.1186/s40733-020-00055-9**

After publication of the original article [1] the authors have noticed that some of the *P*-values were incorrect at time of publication. This correction article is to show the incorrect & correct values and to explain how this affects the original publication.

For Fig. 1, Table 3 and supplementary file 1 only the correct information is available via this correction article.

- **Abstract**
  - ◦ **Correct:** The number of annual antibiotic courses (− 0.7 courses, *p* = 0.04) was significantly reduced in the anti-IL5/IL5R group, and total number of exacerbation events were reduced in both groups (− 4.4 events/year, *p* < 0.05 in the anti-IL5/IL5R group and − 1.5events/year, *p* < 0.05 in the anti-IgE group).
  - ◦ **Incorrect:** The number of annual antibiotic courses (− 0.7 courses, *p* = 0.04) and total number of exacerbation events (− 4.4 events/year, p < 0.05) were reduced in the anti-IL5/IL5R group.
- **Results**
  - ◦ **Correct:** The reduction in total exacerbation events was statistically significant in the anti-IgE group (from 4.7/year to 3.2/year, *p* = 0.006)
  - ◦ **Incorrect:** The reduction in total exacerbation events was not statistically significant in the anti-IgE group (from 4.7/year to 3.2/year, *p* = 0.229)
- Discussion
  - ◦ **Correct:** This study suggests that anti-IL5/IL5R and anti-IgE therapies reduce per oral glucocorticoid use and total exacerbations.
  - ◦ **Incorrect:** This study suggests that anti-IL5/IL5R therapy reduces exacerbations and anti-IL5/IL5R and anti-IgE therapies reduce per oral glucocorticoid use.
  - ◦ **Correct:** Results of this study are otherwise in concordance with previous studies, except from the lack of statistical significance in the reduction of the daily OCS dose with omalizumab.
  - ◦ **Incorrect:** Results of this study are otherwise in concordance with previous studies, except from the lack of statistical significance in the reduction of the daily OCS dose and total annual exacerbation events with omalizumab.
- Conclusions
  - ◦ **Correct:** In this retrospective real-life study, biologicals reduced OCS courses and total exacerbation events, and CRS-surgery rate in patients with co-morbid CRS.
  - ◦ **Incorrect:** In this retrospective real-life study, biologicals reduced OCS courses in both groups and reduced total exacerbation events in the anti-IL5/IL5R group, and CRS-surgery rate in patients with co-morbid CRS.

**Fig. 1:**
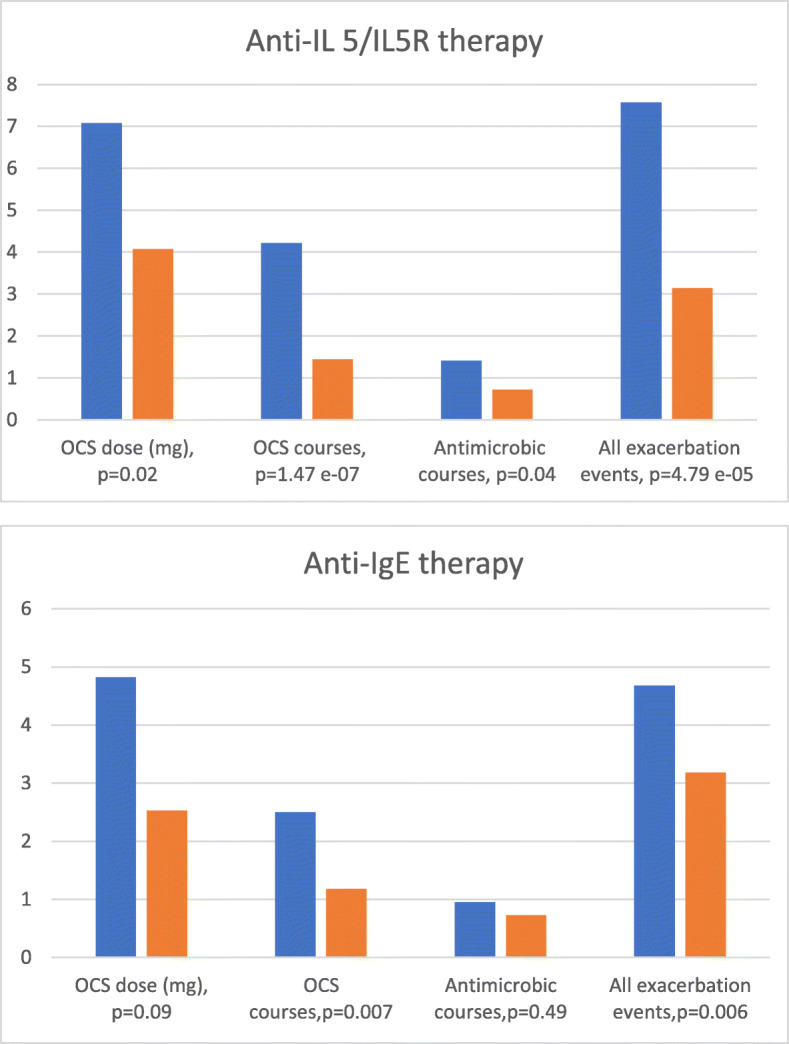
The corrected *P*-value is shown for All exacerbation events in Anti-IgE therapy

**Table 3:** corrected *P*-values for Sick leaves, Total number of exacerbation events and Mean FEV1 (litres)

	Anti-IL5/IL5R (reslizumab, mepolizumab, benralizumab)	*N* = 42		Anti-IgE (omalizumab)	*N* = 22	
	Baseline	Change	P	Baseline	Change	P
Sick leaves **	0.93 (0–6. SD 1.72)	−0.58 (−6–6. SD 1.86)	0.037	0.59 (0–4. SD 1.03)	−0.14 (−3–11. SD 1.18)	0.602
Total number of exacerbation events ***	7.57 (1–26. SD 5.59)	−4.43 (−22–13. SD 6.24)	4.79 e^− 05^	4.68 (0–11. SD 3.40)	−1.50 (− 7–21. SD 5.55)	0.006
Mean FEV1 (litres)	2.27 (1.0–4.2. SD 0.78)	0.17 (− 0.5–1.3. SD 0.41)	0.317	2.83 (1.5–4.2. SD 0.86)	0.05 (− 0.6–1.3. SD 0.54)	0.720

** Number due to asthma during the last 12 months

*** defined as the sum of courses of oral glucocorticoid and antimicrobial drugs, sick leaves, hospitalisations and emergency room visits due to asthma during the last 12 months

The total number of exacerbation events was statistically significantly reduced in both anti-IL5/IL5R and anti-IgE groups. The reduction of the number of sick leaves was significant in patients receiving anti-IL5/IL5R therapy, but not in patients receiving anti-IgE therapy. The improvement of lung function measured by FEV1 was not significant in either of the therapy groups.

In this retrospective real-life study, biologicals reduced OCS courses in both groups and reduced total number of exacerbation events in both the anti-IL5/IL5R group and in the anti-IgE group. After the correction our conclusions remain: biological therapy reduces exacerbations and per oral corticosteroid use.

## Supplementary information

**Additional file 1.**
