# Supplementary material for: Correction to: A comparison of biologicals in the treatment of adults with severe asthma – real-life experiences
Source: Asthma Res Pract. 2020 Oct 2;6:10. doi: 10.1186/s40733-020-00063-9 (PMC7532111; doi:10.1186/s40733-020-00063-9)
Supplement: Supplementary file 1 — Additional file 1. [file 40733_2020_63_MOESM1_ESM.docx]

**Additional file 1**

**Table 1**.

| Characteristics | Reslizumab, n=13 | Mepolizumab, n=24 | Benralizumab, n=5 | Omalizumab, n=22 |
| --- | --- | --- | --- | --- |
| Time of use (months) | 15.3 (4-27. SD 7.79) | 12.25 (3-31. SD 9.10) | 8.8 (5-12. SD2.79) | 49.41 (6-127. SD 35.34) |
| Age (years) | 56 (39-72. SD 10.13) | 58 (32-72. SD 9.14) | 48 (39-55. SD 6.97) | 48 (28-76. SD 10.67) |
| Women (number, (%)) | 6 (46) | 14 (58) | 3 (60) | 15 (68) |
| Smokers (number, (%)) | 0 (0) | 0 (0) | 0 (0) | 0 (0) |
| Ex-smokers (number, (%)) | 4 (31) | 6 (25) | 2 (40) | 2 (9) |
| Body mass index (kg/m^2^) | 25.5 (20.8-34.1. SD 3.2) | 30.7 (16.9-39.0. SD 5.4) | 29.7 (26.0-36.1. SD 3.7) | 27.7 (20.4-36.8. SD 4.1) |
| Nasal polyposis (number, (%)) | 12 (92) | 16 (67) | 3 (60) | 12 (55) |
| Chronic rhinosinusitis (CRSwNP or CRSsNP) (number, (%)) | 13 (100) | 22 (92) | 4 (80) | 18 (82) |
| Allergic rhinitis (number, (%)) | 4 (31) | 6 (25) | 2 (40) | 17 (77) |
| ASA intolerance (number, (%)) | 2 (15) | 6 (25) | 1 (20) | 5 (23) |
| Osteoporosis (number, (%)) | 6 (46) | 10 (42) | 0 (0) | 8 (36) |
| Hypertension (number, (%)) | 3 (23) | 14 (58) | 3 (60) | 8 (36) |
| Diabetes mellitus (number, (%)) | 0 (0) | 3 (13) | 0 (0) | 3 (14) |
| Hypothyroidism (number, (%)) | 0 (0) | 6 (25) | 1 (20) | 3 (14) |
| Coronary artery disease (number, (%)) | 0 (0) | 1 (4) | 0 (0) | 0 (0) |
| Gastroesophageal reflux disease (number, (%)) | 2 (15) | 7 (29) | 1 (20) | 10 (45) |
| Atrial fibrillation (number, (%)) | 0 (0) | 3 (13) | 0 (0) | 1 (5) |
| Positive skin prick test or elevated allergen specific serum IgE (number, (%)) | 5 (38) | 9 (38) | 3 (60) | 22 (100) |
| Pathological HRCT findings (number, (%)) * | 11 (85) | 17 (71) | 2 (40) | 14 (64) |
| Patients with daily use of OCS (number, (%)) ** | 9 (69) | 16 (67) | 5 (100) | 10 (45) |
| Mean daily OCS dose before treatment (mg) | 6.69 (0-20. SD 5.70) | 6.98 (0-40. SD 8.36) | 8.50 (5-10. SD 2.00) | 4.82 (0-20. SD 6.50) |
| Courses of OCS before treatment *** | 4 (1-7. SD 1.84) | 4.33 (0-8. SD 2.70) | 4.20 (0-12. SD 4.40) | 2.50 (0-6. SD 1.56) |
| Courses of antibiotics before treatment *** | 2.08 (0-5. SD 1.49) | 1.17 (0-5. SD 1.28) | 0.80 (0-3. SD 1.17) | 0.95 (0-3. SD 0.93) |
| Emergency room visits before treatment *** | 1 (0-8. SD 2.20) | 0.50 (0-5. SD 1.08) | 0 (0-0, SD 0) | 0.18 (0-1. SD 0.39) |
| Sick leaves before treatment *** | 0.85 (0-4. SD 1.56) | 1.13 (0-6. SD 1.92) | 0.20 (0-1. SD 0.40) | 0.59 (0-4. SD 1.03) |
| Hospitalisations before treatment *** | 0.92 (0-8. SD 2.16) | 0.42 (0-4. SD 0.91) | 0 (0-0, SD 0) | 0.45 (0-2. SD 0.66) |
| FEV1 (mean) before treatment (litres) | 2.65 (0.9-4.2. SD 0.82) | 2.00 (1.2-3.2. SD 0.62) | 2.71 (1.7-3.9. SD 0.93) | 2.83 (1.5-4.2. SD 0.86) |
| FVC (mean) before treatment (litres) | 3.72 (1.7-5.3. SD 1.07) | 3.16 (1.7-5.1. SD 0.85) | 3.84 (2.2-6.00. SD 1.29) | 3.82 (2.1-5.8. SD 0.85) |
| FEV1/FVC (mean) before treatment | 0.71 (0.51-0.81. SD 0.10) | 0.64 (0.47-0.91. SD 0.13) | 0.74 (0.69-0.79. SD 0.04) | 0.74 (0.48-0.91. SD 0.13) |
| Blood eosinophil count (mean) before treatment (E9/litre) | 0.49 (0-1.84. SD 0.46) | 0.48 (0.03-1,09. SD 0.32) | 0.23 (0-0.44. SD 0.19) | 0.43 (0-1.34. SD 0.41) |
| Exhaled nitric oxide (mean) (ppb) **** | 29.97 (5-110. SD 37.05) | 27.6 (5-107. SD 25.61) | 20.9 | 35.66 (5-123. SD 36.30) |
| Serum total IgE (mean) (kU/litre) | 571.27 (33-4627. SD 1304.72) | 152.89 (8-404. SD 133.86) | 63.00 (47-79. SD 16.00) | 313.36 (14-720. SD 248.76) |
| ACT (mean) ***** | 17.63 (10-24. SD 4.58) | 14.76 (8-23. SD 3.79) | 14.33 (9-18. SD 3.86) | 13.4 (7-22. SD 4.82) |

*defined as HRCT (high resolution computed tomography) detected ground glass pattern, bronchiectasis, mucous plugs, atelectasis, nodularity or bronchial thickening

** OCS= per oral corticosteroids

*** Number due to asthma during the past 12 months before biological treatment initiation

**** In the benralizumab group, data on exhaled nitric oxide was available only in one patient.

***** ACT = Asthma control test, maximal score 25 points

Clinical characteristics and co-morbidity of asthma patients before reslizumab, mepolizumab, benralizumab and omalizumab therapy. (Additional file)

**Table 2.**

| **Gender (M=male, F=female)** | **Age (years)** | **Adverse effect / No response** | **Severity of adverse effect** | **Treatment** | **Intervention** |
| --- | --- | --- | --- | --- | --- |
| F | 50 | Rise in transaminases, transient limb pain after injection | 2 | Reslizumab | Discontinuation of therapy at 5 months |
| M | 41 | No response to therapy | No adverse effect | Reslizumab | Discontinuation of therapy at 6 months |
| F | 53 | Transient limb joint rigidity after injection | 1 | Reslizumab | No intervention |
| M | 72 | Recurrent gouts, oral reaction with blisters and lip swelling 3 weeks from injection | 2 | Mepolizumab | Discontinuation of therapy at 7 months |
| F | 49 | No response to therapy | No adverse effect | Mepolizumab | Discontinuation of therapy at 7 months |
| M | 65 | No response to therapy | No adverse effect | Mepolizumab | Discontinuation of therapy at 13 months |
| M | 50 | Pneumopleuritis and pericarditis during biological therapy | 3 | Mepolizumab | Discontinuation of therapy at 4 months |
| F | 32 | Recurrent infections, no response to therapy | No adverse effect | Mepolizumab | Discontinuation of therapy at 3 months |
| M | 51 | No response to therapy, back pain and fatigue after injection | 2 | Mepolizumab | Discontinuation of therapy at 6 months |
| F | 56 | Headache | 1 | Mepolizumab | No intervention |
| F | 58 | Headache | 1 | Mepolizumab | No intervention |
| F | 51 | No response to therapy | No adverse effect | Benralizumab | Discontinuation of therapy at 5 months |
| F | 52 | Fever after injection | 1 | Benralizumab | No intervention |
| M | 51 | Dyspnoea after injection | 2 | Omalizumab | Discontinuation of therapy at 33 months |
| F | 76 | No response to therapy | No adverse effect | Omalizumab | Discontinuation of therapy at 18 months |
| M | 39 | No response to therapy | No adverse effect | Omalizumab | Discontinuation of therapy at 21 months |
| F | 41 | No response to therapy | No adverse effect | Omalizumab | Discontinuation of therapy at 12 months |
| F | 28 | No response to therapy, pregnancy | No adverse effect | Omalizumab | Discontinuation of therapy at 6 months |
| F | 56 | Fatigue after injection | 1 | Omalizumab | No intervention |
| F | 39 | Transient headache and injection site pain | 1 | Omalizumab | No intervention |
| F | 47 | Worsening of lower limb pitting oedema and nausea after injection | 1 | Omalizumab | No intervention |
|  |  |  |  |  |  |

Adverse events during mepolizumab, reslizumab, benralizumab and omalizumab treatment. The severity is classified at a scale from 1 to 5, where 1 is mild, 2 is moderate, 3 is severe, 4 is life-threatening or disabling and 5 is deaths. (Additional file)

**Table 3.**

| **Controller medication before biologicals** | **Reslizumab, n=13** | **Mepolizumab, n=24** | **Benralizumab, n=5** | **Anti-IL5/IL5R** **therapy** (reslizumab, mepolizumab and benralizumab)**, n=42** | **Omalizumab, n=22** |
| --- | --- | --- | --- | --- | --- |
| **ICS daily dose (number, (%)) *** |  |  |  |  |  |
| **High** | 13 (100) | 24 (100) | 4 (80) | 41 (98) | 20 (91) |
| **Intermediate** | 0 (0) | 0 (0) | 1 (20) | 1 (2) | 2 (9) |
| **Low** | 0 (0) | 0 (0) | 0 (0) | 0 (0) | 0 (0) |
| **Daily use of long acting beta-agonist (number, (%))** | 12 (92) | 24 (100) | 5 (100) | 41 (98) | 19 (86) |
| **Daily use of theophylline (number, (%)) **** | 2 (15) | 4 (17) | 1 (20) | 7 (17) | 7 (32) |
| **Daily use of montelukast (number, (%)) ***** | 11 (85) | 15 (63) | 4 (80) | 30 (71) | 13 (59) |
| **Daily use of long-acting anticholinergic (number, (%))** | 6 (46) | 17 (71) | 4 (80) | 27 (64) | 15 (68) |
| **Daily use of one asthma controller medication in addition to ICS (number, (%))** | 2 (15) | 5 (21) | 0 (0) | 7 (17) | 4 (18) |
| **Daily use of two or more asthma controller medications in addition to ICS (number, (%))** | 11 (85) | 19 (79) | 5 (100) | 35 (83) | 18 (82) |
| **Daily use of OCS (number, (%))** | 9 (69) | 16 (67) | 5 (100) | 30 (71) | 10 (45) |

* ICS=inhaled corticosteroid treatment. For beclomethasone and budesonide: low dose 0-399 µg/day, intermediate dose 400-799 µg/day and high dose 800 µg/day or more, for fluticasone low dose 0-249 µg/day, intermediate dose 250-499 µg/day and high dose 500 µg/day or more, and for ciclesonide low dose 0-159 µg/day, intermediate dose 160-319 µg/day and high dose 320 µg/day or more. The ICS daily dose was defined as the dose prior to initiation of biological therapy.

** Additionally, 3 patients in the reslizumab group, 5 patients in the mepolizumab group and 3 patients in the omalizumab group had received theophylline earlier but discontinued it because of lack of response or side effects.

*** Additionally, 1 patient in the reslizumab group, 3 patients in the mepolizumab group and 6 patients in the omalizumab group had received montelukast earlier but discontinued it because of lack of response or side effects.

Controller medication of asthma patients before reslizumab, mepolizumab, benralizumab and omalizumab treatment. (Additional file)

**Table 4**.

| Imaging findings |  | Anti-IL5/IL5R therapy | Anti-IgE therapy |
| --- | --- | --- | --- |
|  |  | **Reslizumab, Mepolizumab, Benralizumab, n=38** | **Omalizumab, n=17** |
| HRCT* findings | **Atelectasis, (number, (%))** | 11 (29) | 6 (35) |
|  | **Ground glass pattern (number, (%))** | 4 (11) | 3 (18) |
|  | **Nodularity (number, (%))** | 8 (21) | 4 (24) |
|  | **Mucous plugs (number, (%))** | 12 (32) | 4 (24) |
|  | **Bronchial wall thickening (number, (%))** | 22 (58) | 6 (35) |
|  | **Bronchial dilatation (number, (%))** | 9 (24) | 6 (35) |
|  | **Bronchiectasis (number, (%))** | 8 (21) | 1 (6) |
| Sinonasal CT** findings | **Mucous oedema (number, (%))** | 30 (79) | 8 (47) |
|  | **Polyps (number, (%))** | 20 (53) | 5 (29) |
|  | **Air-fluid level (number, (%))** | 4 (11) | 2 (12) |

* High resolution computed tomography (HRCT) of the chest

** Computed tomography

Imaging findings of asthma patients receiving anti-IL5 or anti-IgE therapy. In all but 2 patients the HRCT imaging was done before initiation of biological therapy, in 2 patients the imaging was done after initiating anti-IgE therapy. In all but 2 patients, the sinonasal CT imaging was done before initiation of biological therapy, in 2 patients the imaging was done after initiating anti-IgE therapy. (Additional file

**Table 5.**

|  | Reslizumab | N=13 |  | Mepolizumab | N=24 |  | Benralizumab | N=5 |  | Omalizumab | N=22 |  |
| --- | --- | --- | --- | --- | --- | --- | --- | --- | --- | --- | --- | --- |
|  | **Baseline** | **Change** | **P** | **Baseline** | **Change** | **P** | **Baseline** | **Change** | **P** | **Baseline** | **Change** | **P** |
| Mean daily OCS dose (mg) * | 6.69 (0-20. SD 5.70) | -3.19 (-10-10. SD 5.11) | 0.051 | 6.98 (0-40. SD 8.36) | -2.69 (-35-25. SD 9.63) | 0.193 | 8.50 (5-10. SD 2.00) | -4.00 (-10-0. SD 3.39) | 0.078 | 4.82 (0-20. SD 6.50) | -2.29 (-20-0. SD 5.80) | 0.085 |
| Courses of OCS ** | 4.00 (1-7. SD 1.84) | -2.92 (-7-1. SD 2.40) | 0.001 | 4.33 (0-8. SD 2.70) | -2.86 (-8-4.2. SD 2.73) | 4.34 e^-05^ | 4.20 (0-12. SD 4.40) | -1.97 (-7.64-3.8. SD 3.83) | 0.362 | 2.50 (0-6. SD 1.56) | -1.32 (-4-10. SD 2.01) | 0.007 |
| Courses of antibiotics ** | 2.08 (0-5. SD 1.49) | -1.54 (-5-4. SD 2.24) | 0.034 | 1.17 (0-5. SD 1.28) | -0.35 (-3-7. SD 1.97) | 0.409 | 0.80 (0-3. SD 1.17) | -0.12 (-1.8-2.18. SD 1.34 | 0.862 | 0.95 (0-3. SD 0.93) | -0.23 (-3-5. SD 1.47) | 0.488 |
| Emergency room visits ** | 1 (0-8. SD 2.20) | -0.85 (-6-0. SD 1.75) | 0.119 | 0.50 (0-5. SD 1.08) | -0.05 (-2-4. SD 1.02) | 0.816 | 0 (0-0, SD 0) | 0 |  | 0.18 (0-1. SD 0.39) | 0.18 (-1-5. SD 1.19) | 0.492 |
| Hospitalisations** | 0.92 (0-8. SD 2.16) | -0.85 (-8-1. SD 2.21) | 0.210 | 0.42 (0-4. SD 0.91) | 0.12 (-2-4. SD 1.30) | 0.670 | 0 (0-0, SD 0) | 0 |  | 0.45 (0-2. SD 0.66) | 0.00 (-1-5. SD 1.21) | 1.00 |
| Sick leaves ** | 0.85 (0-4. SD 1.56) | -0.85 (-4-0. SD 1.56) | 0.085 | 1.13 (0-6. SD 1.92) | -0.51 (-6-6. SD 2.15) | 0,264 | 0.20 (0-1. SD 0.40) | -0.2 (-1-0. SD 0.4) | 0.374 | 0.59 (0-4. SD 1.03) | -0.14 (-3-11. SD 1.18) | 0.602 |
| Total number of exacerbation events *** | 8.92 (2-18. SD 6.70) | -7.08 (-22-5. SD 7.41) | 0.006 | 7.33 (1-17. SD 4.86) | -3.44 (-17-13. SD 5.46) | 0.006 | 5.2 (0-13. SD 4.66) | -2.29 (-6.45-3.8. SD 3.72) | 0.286 | 4.68 (0-11. SD 3.40) | -1.50 (-7-21. SD 5.55) | 0.006 |
| Blood eosinophil count (E9/litre) | 0.49 (0-1.84. SD 0.46) | -0.48 (-1.79-0.01. SD 0.47) | 0.007 | 0.48 (0.03-1,09. SD 0.32) | -0.39 (-1.04-(-0.03. SD 0.33) | 2.19 e^-05^ | 0.23 (0-0.44. SD 0.19) | -0.24 (-0.44-0.15. SD 0.28) | 0.342 | 0.43 (0-1.34. SD 0.41) | -0.08 (-0.61-0.52. SD 0.41) | 0.433 |
| Mean FEV1 (litres) | 2.65 (0.9-4.2. SD 0.82) | 0.30 (-0.28-1.28. SD 0.42) | 0.035 | 2.00 (1.2-3.2. SD 0.62) | 0.13 (-0.5-1.2. SD 0.41) | 0.147 | 2.71 (1.7-3.9. SD 0.93) | -0.005 (-0.3-0.3. SD 0.21) | 0.970 | 2.83 (1.5-4.2. SD 0.86) | 0.05 (-0.6-1.3. SD 0.54) | 0.720 |
| ACT (mean) **** | 17.63 (10-24. SD 4.58) | 4.60 (-1-13. SD 5.89) | 0.044 | 14.76 (8-23. SD 3.79) | 4.31 (-15-12. SD 5.94) | 0.013 | 14.33 (9-18. SD 3.86) | 5.00 (1-9. SD 4.00) | 0.430 | 13.40 (7-22. SD 4.82) | 8.50 (2-17. SD 5.63) | 0.015 |

*OCS = per oral corticosteroid

** Number due to asthma during the past 12 months or during follow-up

*** defined as the sum of courses of oral glucocorticoid and antimicrobial drugs, sick leaves, hospitalisations and emergency room visits due to asthma during the last 12 months

**** ACT = Asthma control test, maximal score 25 points

Treatment response in asthma patients with reslizumab, mepolizumab, benralizumab and omalizumab treatment. Patients with discontinued biological therapy were included in the analyses. (Additional file)

**Table 6.**

| Characteristics prior to biological therapy | Anti-IL5/IL5R therapy |  |  | Anti-IgE therapy |
| --- | --- | --- | --- | --- |
|  | **Reslizumab, n=13** | **Mepolizumab, n=20** | **Benralizumab, n=5** | **Omalizumab, n=17** |
| Asthma (number, (%)) | 13 (100) | 20 (100) | 5 (100) | 17 (100) |
| Nasal polyposis as only indication for biological therapy (number, (%)) | 1 (8) | 2 (10) | 0 (0) | 0 (0) |
| Time of use (months) * | 14.8 (5-27. SD 6.4) | 12.1 (1-29. SD 8.5) | 7.6 (4-11. SD 2.7) | 43.8 (5-109. SD 35.6) |
| Age at analysis (years) | 54.1 (39-72. SD 11.2) | 55.8 (32-71. SD 10.8) | 48.4 (39-55. SD 7.0) | 47.6 (28-76. SD 11.5) |
| Women (number, (%)) | 5 (38) | 10 (50) | 3 (60) | 12 (71) |
| BMI** (kg/m2) | 25.7 (20.9-33.2. SD 2.9) | 29.2 (16.9-38.8. SD 5.4) | 29.7 (26.0-36.1. SD 3.7) | 27.6 (23-35. SD 3.3) |
| Use of intranasal corticosteroids (number, (%)) | 11 (85) | 19 (95) | 4 (80) | 14 (82) |
| Use of oral antihistamine (number, (%)) | 4 (31) | 7 (35) | 2 (40) | 8 (47) |
| Use of montelukast (number, (%)) | 10 (77) | 13 (65) | 4 (80) | 9 (53) |
| Positive skin prick test or allergen specific serum IgE (number, (%)) | 5 (38) | 8 (40) | 3 (60) | 17 (100) |
| Smokers (number, (%)) | 0 (0) | 1 (5) | 0 (0) | 0 (0) |
| Ex-smokers*** (number, (%)) | 2 (15) | 6 (30) | 2 (40) | 2 (12) |
| Nasal polyposis (number, (%)) | 12 (92) | 14 (70) | 3 (60) | 11 (65) |
| Chronic rhinosinusitis (CRSwNP or CRSsNP) (number, (%)) | 11 (85) | 19 (95) | 4 (80) | 15 (88) |
| ASA intolerance (number, (%)) | 2 (15) | 6 (30) | 1 (20) | 3 (18) |
| Osteoporosis (number, (%)) | 5 (38) | 7 (35) | 0 (0) | 4 (24) |
| Blood eosinophil count (E9/l) | 0.51 (0-1.84. SD 0.45) | 0.52 (0.1-1.09. SD 0.33) | 0.23 (0-0.44. SD 0.19) | 0.50 (0.01-1.21. SD 0.42) |
|  |  |  |  |  |
|  |  |  |  |  |

* Time of use of biological therapy

** BMI = Body mass index

*** Defined as previous daily smoking at minimum 1 year

Upper respiratory tract and other characteristics before initiation of reslizumab, mepolizumab, benralizumab or omalizumab treatment. The time of use describes mean time of use of biological therapy in months. (Additional file)
